# Effectiveness and safety of self-pulling and latter transection reconstruction in totally laparoscopic right hemicolectomy

**DOI:** 10.3389/fonc.2024.1320508

**Published:** 2024-01-25

**Authors:** Fuyu Yang, Fan He, Chenglin Tang, Defei Chen, Junjie Xiong, Yu Zou, Saed Woraikat, Kun Qian, Hui Li

**Affiliations:** Department of Gastrointestinal Surgery, The First Affiliated Hospital of Chongqing Medical University, Chongqing, China

**Keywords:** totally laparoscopic right hemicolectomy, right colon cancer, self-pulling and latter transection, laparoscopy-assisted right hemicolectomy, intracorporeal anastomosis, extracorporeal anastomosis

## Abstract

**Background:**

Laparoscopic right hemicolectomy is a standard treatment modality for right colon cancer. However, performing intracorporeal anastomosis (IA) for totally laparoscopic right hemicolectomy (TLRH) remains a challenge for some surgeons. To simplify IA in TLRH we used self-pulling and latter transection (SPLT) reconstruction in TLRH, and compared this procedure with overlap IA and laparoscopy-assisted right hemicolectomy (LARH) in order to evaluate its safety and effectiveness.

**Methods:**

Patients with right colon cancer who underwent SPLT-TLRH, TLRH with overlap IA or LARH between July 2019 and June 2023 were evaluated retrospectively. Basic information, oncological features, perioperative outcomes, and postoperative complications were compared between groups.

**Results:**

In total, 188 patients with right colon cancer that underwent SPLT-TLRH (n = 60), TLRH(n=21) or LARH (n = 107) were included in the study. No patient required conversion to open surgery. The operation time in SPLT-TLRH group was significantly shorter than that in TLRH group (P<0.05). Compared with LARH group, SPLT-TLRH group had significantly longer distal margins, shorter skin incisions (P < 0.001), time to first flatus, time to first defecation, and postoperative hospital stays (P<0.05).

**Conclusion:**

We introduced SPLT to TLRH. The SPLT-TLRH group demonstrated better short-term outcomes. Therefore, we believe that SPLT reconstruction is effective and safe in TLRH for right colon cancer, and can simplify reconstruction.

## 1 Introduction

Laparoscopic colectomy, a standard treatment modality for colon cancer, was first reported in 1991 (1). Since then, it has been favored by many gastrointestinal surgeons because of faster recovery, less trauma, and better short-term outcomes than open colectomy (2, 3). However, with the help of increasingly advanced anastomotic techniques and staplers, surgeons are no longer satisfied with laparoscopy-assisted colectomy, and total laparoscopic colectomy is being developed (4) to further minimize surgical trauma. Although the difficulty of performing intracorporeal anastomosis (IA) for total laparoscopic right hemicolectomy (TLRH) has reduced, the procedure remains challenging for some surgeons, thus hindering the wide use of TLRH in clinical practice (5, 6).

With the concerted efforts of many surgeons, complications after laparoscopic gastrointestinal surgery have declined, however, unsatisfactory outcomes that may be related to the failure of anastomosis and reconstruction of the digestive tract remain (7). Furthermore, in addition to anastomosis (including end-to-side anastomosis, side-to-side anastomosis, and end-to-end anastomosis), the anastomosis modality (including IA and extracorporeal anastomosis [EA]) also affects short-term outcomes after laparoscopic right hemicolectomy. In laparoscopic right colectomy, IA can shorten the length of stay and accelerate the recovery of intestinal function compared with EA (7, 8), however, because of its lower difficulty, EA is more widely used in the clinic. In IA, all intestinal resection and reconstruction are performed intraperitoneally, and the specimen is removed through a smaller incision compared to that used in EA, which is the most critical and difficult step in TLRH. However, because of the need to open the intestinal tract inside the abdominal cavity during IA, it is difficult for surgeons to avoid intestinal content leakage and contact of the stapler with the intra-abdominal tissue, which may increase the risk of abdominal infection. In addition, the inherent difficulty of performing IA deters some surgeons.

Traditional IA of TLRH leaves a defect, the surgeon needs to close the defect with cartridge or barbed suture. We wanted to explore a way to solve the problem that cartridges would increase patient costs and barbed suture would increase operation time. We were inspired by the good results of Self-pulling and latter transection (SPLT) reconstruction in total laparoscopic total gastrectomy and total laparoscopic distal gastrectomy (9, 10). SPLT reconstruction can reduce the difficulty of anastomosis and save stapler cartridges (10), however, it has not been used for TLRH. Combined with the low average income of the Chinese people and the basic national conditions of China’s diagnosis related groups payment, surgeons cannot simplify TLRH by using a more cartridge to close the defect, so we used SPLT reconstruction in TLRH. In this study, SPLT reconstruction in TLRH was described and compared with overlap IA and laparoscopy-assisted right hemicolectomy (LARH), in order to evaluate its safety and effectiveness.

## 2 Patients and methods

### 2.1 Patients

We enrolled consecutive patients with right colon cancer who underwent SPLT-TLRH, TLRH with overlap IA or LARH at the First Affiliated Hospital of Chongqing Medical University between July 2019 and June 2023. The inclusion criteria were as follows: patients with malignant tumors of the appendix, cecum, ascending colon, or hepatic flexure of the colon diagnosed before surgery, and patients who underwent TLRH or LARH. Patients who underwent resection of other organs and were scheduled for simultaneous intra-abdominal surgery, patients who underwent emergency surgery, and patients with anastomosis not belonging to one of SPLT reconstruction, overlap IA or EA were excluded. All patients were informed of the three surgical methods and their advantages and disadvantages, and the corresponding informed consent was signed after the patients chose the surgical method. Patients are grouped by SPLT-TLRH, TLRH and LARH per the three types of anastomosis, SPLT reconstruction, overlap IA and EA, respectively. After preoperative evaluation, we would perform D2 lymphadenectomy for cT1 patients and D3 lymphadenectomy for cT2-4 patients. Basic information (sex, age, main complications, preoperative hemoglobin, and preoperative albumin, etc.), oncological features (tumor stage, proximal margin, distal margin, and number of lymph nodes collected, etc.), perioperative outcomes (length of skin incision, postoperative hemoglobin, postoperative albumin, and recovery of postoperative intestinal function, etc.), and postoperative complications (abdominal infection, anastomotic leakage, and anastomotic bleeding, etc.) were compared between the SPLT-TLRH group and TLRH group, SPLT-TLRH group and LARH group, respectively. Written informed consent was obtained from all patients.

### 2.2 Procedures of LARH and EA

After induction of general anesthesia, the patient took the supine split-leg position and spread their legs about 45 degrees. A curved incision under the navel was made, and a 10-mm trocar for the cameraman was placed. After the establishment of pneumoperitoneum, a 12-mm trocar was placed in the left upper abdomen and left lower ventral side, and a 5-mm trocar was placed in the right lower abdomen and left lower ventral side, for the surgeon and assistant, respectively. The surgeon stood between the patient’s legs, the assistant was on the patient’s left side, and a photographer was outside the patient’s left thigh. After separating the mesentery from the small intestine and exposing the intestine, a midline upper abdominal incision approximately 6 cm in length was made, and the right colon of the patient was pulled out of the abdomen. Transection of the middle part of the transverse colon and 15 cm from the end of the ileum was performed *in vitro*, and a 5 mm incision was made on the antimesenteric side at a distance of 7 cm from both sides. After disinfecting the incision, a linear cutting stapler (REACH IM60AM) was placed, a side-to-side anastomosis of the antimesenteric side was performed, and the common opening was closed with two cartridges.

### 2.3 Procedures of overlap IA for TLRH

The small intestine 15cm from the end of the ileum and the middle transverse colon were cut off by a linear cutting stapler, respectively. Then the ileum was placed next to the transverse colon isoperistaltic and after ileotomy and colotomy a stapled overlap side-to-side anastomosis was made whereafter the remaining defect is closed by a barbed suture or a linear cutting stapler (**Supplementary Video 1**), and a transverse incision of approximately 4 cm long was made in the middle of the lower abdomen for extracting the specimen.

### 2.4 Procedure of SPLT-TLRH

**Figure 1:**
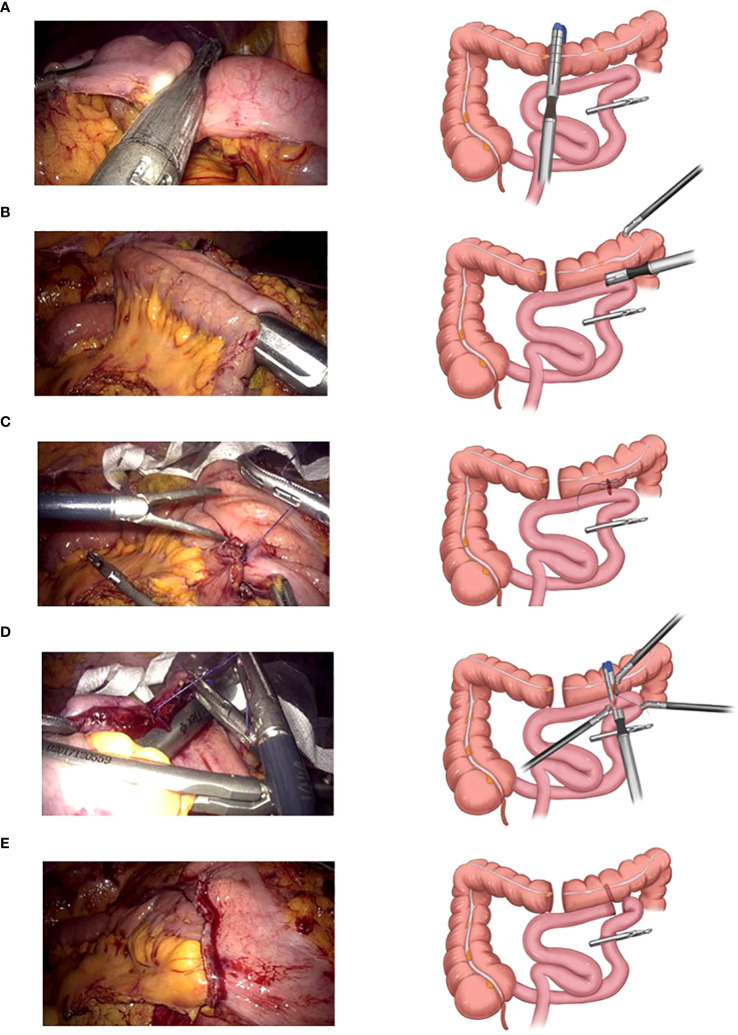
Self-pulling and latter transection (SPLT) reconstruction in totally laparoscopic right hemicolectomy (TLRH). **(A)** The middle transverse colon is cut off. **(B)** The small intestine and colon are anastomosed in an isoperistaltic pattern. **(C)** The middle of the common incision is sutured, leaving 5 cm suture. **(D)** The small intestine is cut off and the common incision is closed simultaneously with only one cartridge. **(E)** The SPLT reconstruction of TLRH is complete.

The basic laparoscopic procedure was the same as that in the LARH group. The small intestine, 15 cm from the end of the ileum, was closed using pug forceps or a sterilized rope. After cutting the middle transverse colon using a linear cutting stapler (**Figure 1A**), the chief surgeon and assistant exchanged positions and overlapped the small intestine and transverse colon. A small incision was made and sterilized 7 cm from the broken end of the transverse colon and at the proximal antimesenteric side of the closed small intestine, and the small intestinal colonic anastomosis was completed in an isoperistaltic pattern (**Figure 1B**). The common incision was sterilized again, and the chief surgeon and assistant swapped positions again. The middle of the common incision was sutured leaving a 5 cm suture (**Figure 1C**), the assistant pulled the suture and assisted the surgeon, through the self-pulling action of the distal small intestine, the small intestine was then cut off, and the common incision was closed simultaneously with a linear cutting stapler (**Figure 1D**). The SPLT reconstruction of the TLRH was complete (**Figure 1E**; **Supplementary Video 2**). The specimen was extracted after an incision similar to overlap IA made.

### 2.5 Postoperative management

All patients in the three groups received standardized postoperative management. All patients were treated with antibiotic prophylaxis after surgery. If no infection was found, antibiotics were discontinued 48 h after the operation. Patients were encouraged to drink water on the first and second day after surgery. If there were no adverse reactions, a small amount of liquid diet was administered one day after drinking. Based on the patient’s condition, a soft diet was introduced 2-3 days after the fluid diet. The patients were encouraged to get out of bed on the second postoperative day, and patients without complications were discharged after receiving total parenteral nutrition.

### 2.6 Follow‐up protocol

Patients were followed-up for 1 month after the operation to assess whether they had any discomfort. Patients with stage I will be recommended for observation, and we suggested postoperative adjuvant chemotherapy for patients with stage II and above. Patients with pathological stage II or higher were recommended tumor markers, history and physical examination every 3–6 months for 2 years, then every 6 months for a total of 5 years. For Patients with stage II or III, chest/abdominal/pelvic contrast-enhanced computerized tomography scan was performed every 6-12 months, and for stage IV, the frequency needs to be increased to once every 3–6 months. Every patient was advised to complete colonoscopy in one year after surgery.

### 2.7 Statistical methods

The SPSS software (version 25.0, IBM Corporation, Armonk, N.Y., USA) was used for statistical analysis. The Kolmogorov-Smirnov test was used to determine the normal distribution of continuous variables. According to the normality of the data continuous variables were represented as median (interquartile range) or mean ± standard deviation, and were tested using the Mann-Whitney U test or Student’s t-test. Sex, American Society of Anesthesiologists (ASA) grade, complications, and other categorical variables were represented as frequencies (%). The chi-square test was used to compare the classified variables. Statistical significance was set at P < 0.05.

## 3 Results

60 patients who underwent SPLT-TLRH, 21 patients who underwent TLRH with overlap IA and 107 patients who underwent LARH with EA were included in this study. We compared the basic characteristics, oncological features, and short-term postoperative outcomes between SPLT reconstruction and overlap IA, SPLT reconstruction and LARH for right colon cancer, respectively. **Table 1** shows the basic information and oncological features of the three groups. There were no significant differences in the basic characteristics of the three groups, including sex, age, body mass index (BMI), smoking, drinking, main comorbidity, preoperative hemoglobin, preoperative albumin, and ASA scores. In terms of oncological characteristics, the distal margin of the SPLT-TLRH group was significantly longer than that of the LARH group [15(11,20) vs. 10(7,13.8), P < 0.001], however, there was no significant differences in the proximal margin, specimens with an inadequate margin, tumor size, tumor differentiation, pT stage, number of positive lymph nodes, number of lymph node collections, metastasis, or TNM stage of the three groups. There were 3 patients with liver metastasis and 1 patient with greater omentum metastasis in SPLT group. In addition, in LARH group, 7 patients had liver metastasis and 2 patients had greater omentum metastasis. Patients with liver metastases underwent partial hepatectomy or radiofrequency ablation after right hemicolectomy, patients with omentum metastases did not need another surgery because the greater omentum had already been removed. The number of patients with lymph node metastasis in the SPLT-TLRH group, TLRH group and LARH group was 16,18,27, respectively.

**Table 1:** Patient characteristics and oncology features.

	SPLT-TLRH(n=60)	TLRH(n=21)	P	LARH(n=107)	P
Age(year)	64.7 ± 13.0	61.6 ± 15.5	0.366	64.2 ± 13.1	0.808
Sex (male/female)	27/33	11/10	0.56	59/48	0.208
BMI (Kg/m2)	22.3 ± 2.5	23.3 ± 2.6	0.12	22.3 ± 3.4	0.888
Smoking	12(20%)	6(28.6%)	0.542	36(33.6%)	0.062
Drinking	12(20%)	5(23.8%)	0.759	25(23.4%)	0.615
Abdominal surgery history	23(38.3%)	9(42.9%)	0.715	26(24.3%)	0.056
Main comorbidity					
T2MD	14(23.3%)	1(4.8%)	1	17(15.9%)	0.235
Hypertension	17(28.3%)	8(38.1%)	0.405	28(26.2%)	0.762
CHD	5(8.3%)	0	0.32	9(8.4%)	0.986
COPD	1(1.7%)	0	1	0	0.359
Preoperative hemoglobin(g/L)	108.3 ± 26.1	109.9 ± 31.4	0.821	112.5 ± 25.7	0.318
Preoperative albumin (g/L)	38.4 ± 5.7	37.0 ± 4.9	0.305	38.2 ± 4.0	0.845
ASA score(I-II/III-IV)	26/34	11/10	0.734	50/57	0.672
Margin (cm)					
Proximal margin	13(10,17)	13(10,17)	0.574	13(11,18)	0.254
Distal margin	15(11,20)	14(11,17)	0.601	10(7,13.8)	<0.001
Specimen with an inadequate margin	4(6.7%)	0	0.568	9(8.4%)	0.772
Tumor size (cm)	4(3,5.3)	3(2.5,4.5)	0.12	4(3,5)	0.932
Tumor differentiation (low/middle/high)	12/42/6	2/16/3	0.514	21/82/4	0.253
pT stage	8/5/33/14	4/2/13/2	0.546	14/11/65/17	0.678
Lymph node					
Positive	0(0,1)	0(0,2)	0.288	0(0,0.5)	0.901
Total removed	17(13.5,23.5)	16(12,19)	0.221	17(14,21.5)	0.703
Distant metastasis	4(6.7%)	0	0.568	9(8.4%)	0.772
TNM stage	11/31/13/4	5/8/8/0	0.284	23/55/20/9	0.91

Variables are presented as n (%), mean ± standard deviation or median (interquartile range). SPLT, self-pulling and latter transection; TLRH, totally laparoscopic right hemicolectomy; LARH, laparoscopy-assisted right hemicolectomy; BMI, body mass index; T2MD, type 2 diabetes mellitus; CHD, coronary atherosclerotic heart disease; COPD, chronic obstructive pulmonary disease; ASA, American Society of Anesthesiologists.

Intraoperative and postoperative data are shown in **Table 2**. None of the 188 patients of the three groups were converted to open surgery, or died within 30 days after the operation. All patients in SPLT-TLRH group and TLRH group completed the corresponding IA successfully. We found that the operative time of SPLT-TLRH group was significantly shorter than the TLRH group [162.5(135,200) vs. 183(150,215) P=0.047]. Compared with the LARH group, the SPLT-TLRH group had earlier time to first flatus [3(2,3) vs. 3(2,4), P =0.016] and defecation [3.5(3,4) vs. 4(3,5), P =0.027], significantly shorter length of skin incisions [4(3,5) vs. 6(5,7), P < 0.001], and shorter postoperative hospital stays [8(7,9) vs. 9(7,11), P=0.037].

**Table 2:** Comparison of postoperative outcomes of both groups.

	SPLT-TLRH(n=60)	TLRH(n=21)	P	LARH(n=107)	P
Operative time(min)	162.5(135,200)	183(150,215)	0.047	180(137.5,215.5)	0.216
Estimated blood loss(mL)	50(30,50)	50(30,50)	0.398	50(30,50)	0.965
Length of skin incision(cm)	4(3,5)	4(3,5)	0.287	6(5,7)	<0.001
Postoperative hemoglobin(g/L)	102.1 ± 19.4	101.4 ± 22.2	0.904	105.2 ± 21.6	0.356
Postoperative albumin (g/L)	32.0 ± 4.2	32.6 ± 4.7	0.543	31.3 ± 4.3	0.335
Intraoperative blood transfusion	3(5%)	0	0.564	3(2.8%)	0.668
Admitted to ICU	3(6%)	1(4.8%)	1	3(2.8%)	0.668
Days of antibiotics use (day)	2(1,6.5)	2(1,6)	0.643	1(1,7.5)	0.986
Rescue analgesia	19(31.6%)	9(42.9%)	0.353	37(34.6%)	0.702
Reoperation	0	0	–	2(1.9%)	1
Time to first flatus (day)	3(2,3)	2(2,3)	0.619	3(2,4)	0.016
Time to first defecation (day)	3.5(3,4)	3(2,4)	0.449	4(3,5)	0.027
Time to ambulation (day)	3(2,4)	3(2,3)	0.146	3(2,4)	0.308
Time to first nutritional powder intake (day)	2(2,2)	2(2,2)	0.081	2(2,2)	0.059
Time to drainage tube removal(day)	7(6,7)	6(5.5,7)	0.39	7(6,8)	0.574
Postoperative hospital stays (day)	8(7,9)	7(6,9)	0.145	9(7,11)	0.037
Unplanned readmissions	2(3.3%)	0	1	4(3.5%)	1
Death	0	0	–	0	–
Hospitalization expenses	69092(60871,78581)	68322(59182,73538)	0.821	71393(63326,85014)	0.139
Postoperative complications					
Abdominal infection	5	3(14.3%)	0.421	11(10.3%)	0.682
Anastomotic leakage	0	0	–	3(2.8%)	0.554
Anastomotic bleeding	1(1.7%)	0	1	2(1.9%)	1
Lymphorrhea	2(3.3%)	0	1	2(1.9%)	0.619
Intestinal obstruction	1(1.7%)	1(4.8%)	0.454	1(0.9%)	1
Incision infection	1(1.7%)	0	1	0	0.359
Pneumonia	1(1.7%)	0	1	5(4.7%)	0.421

Variables are presented as n (%), mean ± standard deviation or median (interquartile range). ICU, intensive care unit.

There were no significant differences between the three groups in terms of estimated blood loss, postoperative hemoglobin, postoperative albumin, intraoperative blood transfusion, ICU admission, days of antibiotics use, rescue analgesia, time to ambulation, time to first nutritional powder intake, time to drainage tube removal, hospitalization expenses or postoperative complications. 7,6 and 26 patients in the SPLT group, TLRH group and LARH group were not placed drainage tube, respectively. There was no reoperation in the SPLT-TLRH group, but two patients were readmitted because of intestinal obstruction and abdominal abscess, respectively. In the LARH group, two patients underwent reoperation because of anastomotic bleeding and anastomotic leakage, three patients were readmitted for intestinal obstruction, and one patient was readmitted for abdominal abscess. There was no reoperation and unplanned readmission in TLRH group. There was no patient received neoadjuvant chemotherapy in this study.

In addition, no other postoperative discomfort and death were reported in the three groups at a one-month follow-up after discharge.

## 4 Discussion

In SPLT reconstruction, the intestines are not cut off temporarily but are ligated with pug forceps or a sterilized rope during gastrointestinal anastomosis. Under the action of self-pulling of the unsevered intestines, the common opening after gastrointestinal anastomosis is closed, while the small intestine not previously severed was cut off (10). This approach was first proposed by Hong et al. in 2016 (11). In this study, SPLT reconstruction, previously used for total laparoscopic gastrectomy, was pioneered and applied to TLRH for treating right colon cancer. Through the self-pulling action of the small intestine, the proximal small intestine and distal colon were overlapped and anastomosed in an isoperistaltic pattern, and a linear cutting stapler was used to cut off the small intestine while closing the common opening of after the enterocolostomy. Traditional IA in total laparoscopic colectomy include functional end-to-end anastomosis (12) and overlap anastomosis (13). Functional end-to-end anastomosis, also known as side-to-side anastomosis, is performed after severing the transverse colon and the small intestine, the two broken intestinal tubes are moved close with anti-peristaltic pattern, and a small incision is made in the ileum and transverse colon, respectively. A linear cutting pattern is then used to anastomose them side-to-side, and the common opening was closed with a barbed suture. Overlap IA as mentioned above, after the side-to-side isoperistaltic anastomosis of the ileum and colon, the intestinal opening was closed with a barbed suture. Both types of IA require intra-abdominal suture carefully, which increases the duration and difficulty of the operation, however, SPLT reconstruction can simultaneously close the remaining defect while severing the small intestine, so it can simplify IA. In addition, it belongs to isoperistaltic anastomosis.

In addition to the aforementioned extracorporeal side-to-side anastomosis, LARH also includes tubular anastomosis (14). Tubular anastomosis refers to end-to-side anastomosis of the ileum and transverse colon by a tubular stapler after transection of the transverse colon *in vitro*, and then using a linear cutting stapler to cut off the distal ileum. Xia et al. reported that the total cost of hospitalization for tubular anastomosis was higher than that for modified side-to-side anastomosis (14), however, tubular anastomosis belongs to isoperistaltic anastomosis. These two types of EA have their own advantages and disadvantages.

In this study, we compared the differences between SPLT construction and overlap IA, SPLT construction and EA in laparoscopic right colon cancer surgery, respectively. We found that the operative time was significantly shorter in the SPLT-TLRH group than in the TLRH group. Because in the overlap anastomosis, the remaining defect needs to be closed by a barbed suture carefully, which takes more time. Compare with the LARH group, the distal margin was significantly longer in the SPLT-TLRH group which is consistent with the results of two previous studies on IA of TLRH (15, 16). This may be because EA needs to pull the intestines out of the abdominal cavity for anastomosis, which will cause the transverse colon in the abdominal cavity to be unable to participate in the anastomosis, while the small intestine has a greater range of motion, therefore, the proximal margins of the two groups are similar. However, the incidence of inadequate margin (<4.2cm) was similar. This is because although the distal margin of the LARH group is shorter, most of them meet the basic requirements. We also found that both the time to first flatus and time to first defecation after SPLT-TLRH were significantly shorter than those after LARH, and the length of hospital stay was significantly shorter. The time to first flatus and time to first defecation are effective indicators for evaluating recovery of intestinal function after colon cancer surgery. The SPLT-TLRH group had faster intestinal function recovery, which may be related to isoperistaltic anastomosis (13) and less intestinal manipulation and mesenteric traction (6). And faster recovery of intestinal function after operation leads to earlier discharge.

Some studies have found that IA increases the risk of abdominal infection because of the opening of the intestinal tract inside the patient’s abdominal cavity (12). But our study did not find this difference. Opening the intestines is unavoidable during intestinal anastomosis, therefore, surgeons need to reduce the incidence of abdominal infection by careful disinfection and reducing the duration of intestinal opening intraperitoneally during IA. Overlap IA requires close the remaining defect carefully, whereas SPLT reconstruction dose not, so in theory, SPLT reconstruction has a lower risk of abdominal infection because it opens the intestine in the abdominal cavity for a shorter time than overlap IA, but our study does not show this difference.

In addition, SPLT reconstruction also has advantages associated with total laparoscopic surgery, such as avoiding multiple pneumoperitoneum establishment, and smaller incisions, similar results have been reported by other studies (17, 18). Our study found that the length of incision in the SPLT group was significantly shorter than it in the LARH group. Smaller incisions can reduce the incidence of incisional hernia, incision infection, and postoperative wound pain and adhesion (19, 20), which are beneficial for the early recovery of patients. The abdominal incision of TLRH is mainly aimed at removing specimens, therefore, the incision is smaller, and its position and direction are more flexible. The incidence of incisional hernia in midline incisions is higher than that in off-midline incisions (21, 22), so surgeons can choose smaller and off-midline incisions in SPLT-TLRH to reduce the incidence of incisional hernia in TLRH. In addition, total laparoscopic surgery can remove the specimen through a natural cavity, such as the female vagina (4), which echoes the current concept of enhanced recovery after surgery (ERAS).

Based on the above comparison, we believe that SPLT reconstruction has different advantages over the two IA and two EA mentioned earlier, including less operation time, smaller incisions, earlier intestinal function recovery, and earlier discharge. Moreover, the SPLT-TLRH group was not inferior to the TLRH group and the LARH group in terms of postoperative complications, estimated blood loss, length of postoperative hospital stays, and pathological features. Therefore, we believe that using SPLT reconstruction in TLRH is safe and effective.

This study has some limitations that should be acknowledged. First, it may had been more appropriate to use only overlap IA cases as the control group. However, overlap IA has not been widely promoted, and there were fewer cases in our hospital. In addition, LARH is a widely used surgery, and its safety and effectiveness has been recognized and confirmed through high-level research (23, 24). Therefore, we compare overlap IA and LARH with SPLT reconstruction TLRH respectively to evaluate the safety and efficacy of SPLT reconstruction, which we think is reasonable. Second, this was a retrospective study with a small sample size. Therefore, we will continue to use SPLT reconstruction in TLRH, and further evaluate its safety and effectiveness. Third, the purpose of this study was to evaluate the effectiveness and safety of SPLT reconstruction in TLRH, therefore, we only evaluated its short-term outcomes, with a short follow-up time, so long-term results are lacking, and longer follow-up is needed in the future. Fourth, although data from our center show that SPLT-TLRH is feasible, we do not yet have a learning curve, and have not solved the learning problems of surgeons in other institutions.

## 5 Conclusion

In this study, we compared differences in pathological features, surgical safety, and short-term postoperative outcomes between SPLT reconstruction and overlap IA, SPLT reconstruction and LARH for right colon cancer, respectively. The operation time in the SPLT-TLRH group was shorter than that in the TLRH group, and compared with the LARH group, the SPLT-TLRH group had smaller incisions, faster recovery of postoperatively intestinal function, and earlier discharge. The difference in surgical safety was not statistically significant. Therefore, we believe that SPLT reconstruction is effective and safe in TLRH for right colon cancer, it can simplify reconstruction in right colon cancer surgeries. A well-designed prospective study should be performed in the future to validate the safety and efficacy of this reconstruction.

## Data availability statement

The raw data supporting the conclusions of this article will be made available by the authors, without undue reservation.

## Ethics statement

The studies involving humans were approved by Medical Ethics Committee of Chongqing Medical University. The studies were conducted in accordance with the local legislation and institutional requirements. Written informed consent for participation was not required from the participants or the participants’ legal guardians/next of kin in accordance with the national legislation and institutional requirements.

## Author contributions

FY: Conceptualization, Writing – original draft, Writing – review & editing. FH: Conceptualization, Writing – original draft, Writing – review & editing. CT: Data curation, Writing – review & editing. DC: Data curation, Writing – review & editing. JX: Methodology, Writing – review & editing. YZ: Data curation, Writing – review & editing. SW: Methodology, Writing – review & editing. KQ: Conceptualization, Writing – review & editing. HL: Conceptualization, Writing – review & editing.
